# Chicago Medical Society

**Published:** 1866-09

**Authors:** 


### PROCEEDINGS OF MEDICAL SOCIETIES.

### CHICAGO MEDICAL SOCIETY.—AUGUST, 1866.

#### ENCEPHALOID CANCER OF THE SKIN.

Dr. Bogue exhibited a very interesting and unusual specimen of tumor, which he had removed from the left forearm of a patient at the Cook County Hospital.

The patient, a young man, 17 years of age, had received a blow from a canteen on the inside of the forearm, just below the elbow. From the effects of this blow there was soreness and swelling, which slowly developed into a peculiar tumor. This was "shaved off" about a year after receiving the injury.

At the time of entering the hospital, twelve months after the first removal of the tumor, the patient was in the following condition: The physical appearance and general health were most excellent. The tumor was nearly two inches in diameter, and globular in form. The whole growth was readily removed by an elliptical incision, since it was confined entirely to the tissue of the skin.

Dr. Lyman, who had made a microscopic examination of the tumor, stated that it was composed of granular matter, without any peculiar or characteristic cells. He regarded it as the product of encephaloid disease.

### SANITARY REPORT.

Dr. Davis gave an interesting verbal report of the sanitary condition of the city during the month of July.

The first ten days of the month had been uniformly damp, hot and sultry, with frequent showers. For the remainder of the month there had been frequent changes, so that there were at no time more than three successive hot days.

During the prolonged hot weather, cholera morbus and dysentery were very prevalent. Dr. Davis had seen three very severe cases of the former disease, which presented some of the sypmtoms of true cholera, but without collapse and shrivelling of tissues.

There had been an unusual number of cases of continued fever with symptoms of typhus. The attack was more rapid than in ordinary enteric fever. Delirium, pain in the head, rheumatic pain in the joints, constipation, and the peculiar odor of typhus, were the characteristic symptoms. There was great difficulty in modifying the disease favorably by treatment.

Dr. Davis proposed to bring the subject more fully before the society at another meeting.

### CASES OF POISONING BY OPIUM AND BY CORROSIVE SUBLIMATE.

Dr. Wanzer reported a case in which a man had taken ℥ii. of tinct. opii. An hour after the poison had been taken, he succeeded in producing emesis, when he directed constant effusions of cold water over the head. Although the symptoms for a time were very alarming, the patient recovered.

Dr. Wanzer also reported a case, in which a woman had intentionally swallowed a solution containing gr. iii. of bichloride of mercury. Large doses of albumen prevented all dangerous symptoms.

### POISONOUS EFFECTS OF ATROPINE INSTILLED UPON THE CONJUNCTIVA.

Dr. Holmes related two cases in which patients had been brought under the constitutional effects of belladonna by the instillation of a drop every hour of a solution of neutral sulphate of atropine, gr. iv. to the ℥ of water.

The patients were both men about 35 years of age, and in vigorous health, suffering, however, severe pain from superficial ulceration of the cornea.

They resided in different sections of the city, and by coincidence each received his prescription in the same afternoon.

Twenty-four hours after commencing the use of the medicine, Dr. Holmes found the patients had been experiencing for about six hours slight effects of the poison. As the local pain had somewhat subsided, the instillation was continued. At the end of twenty-four hours more, the symptoms had become alarming to the friends. The face of each patient was red; the throat dry and voice husky; there was excessive restlessness and tossing, in one case with a peculiar, spasmodic kicking of the legs. The delirium was wild, but without any of the peculiar and ludicrous actions described by some authors.

The pulse was hard and full, beating about 100 per minute. The urine was abundant, but apparently not excessive in quantity.

After discontinuing the remedy twenty-four hours, the constitutional symptoms had disappeared.

There was no evidence that the patients had used by mistake more of the atropine than had been directed, or that there was any unusual size of the lachrymal puncta to promote the passage of the poison through the nasal ducts.

Dr. Holmes stated that he had prescribed atropine in a very large number of cases, but had never before observed its peculiar effects so marked in so short a time.

He had never deemed it necessary to give adults warning except in regard to the danger of taking it by mistake internally.

To avoid such unpleasant effects of atropine, it had been proposed by Liebreich to place a minute pair of spring forceps upon a fold of the lower lid in order to produce eversion of the punctum from the globe. This would cause the superfluous fluid to flow upon the cheek, instead of the mucous membrane of the ducts and fauces.

The report of these cases induced considerable discussion in reference to the use of belladonna.

Dr. Ross had injected subcutaneously with great benefit a drop of a solution of atropine, a grain to the drachm, for the relief of facial neuralgia in a male patient, 71 years of age. The next day, however, although the pain was relieved, there was great general nervous excitement with involuntary spasmodic motion of the legs.

Dr. Davis had observed marked constitutional effects from three doses of one-fiftieth of a grain of atropine each, given every three hours; also marked but not alarming effects from two subcutaneous injections of one seventy-fifth of a grain of atropine, given at an interval of an hour.

Dr. Davis related a case, in which a druggist's clerk, by mistake, had substituted solid extract for the tincture of belladonna in a prescription written for a child three years old. In this way a grain and a half of solid extract was administered. The effects of the first dose was so alarming, that it was not repeated; so alarming, in fact, that, although Dr. Davis saw the child at once, there was every indication of a fatal result. After very minute and frequent doses of morphine for six hours, the child became perfectly quiet and out of danger.

Dr. Davis had found minute and frequent doses of atropine very efficient in overcoming the unpleasant effects of morphine.

Dr. Ingals stated that he had seen decided constitutional effects in a child, who had taken three drops of the fluid extract of belladonna.

Dr. Nelson had observed the dilatation of the pupil to continue ten days after two instillations on the conjunctiva of two drops each of a solution of atropine, two grains to the ounce.

